# White coat color in Vietnamese native buffalo is attributed to the LINE1 insertion in *ASIP*

**DOI:** 10.1007/s11250-025-04309-7

**Published:** 2025-02-11

**Authors:** Thuy Thanh Nguyen, Quan Viet Le, Van Huu Nguyen, Hai Thanh Duong, Takehito Tsuji

**Affiliations:** 1 Graduate School of Environmental and Life Science, Okayama University, Okayama, Japan; 2 Faculty of Animal Sciences and Veterinary Medicine, University of Agriculture and Forestry, Hue University, Hue, Vietnam; 3 Graduate School of Environmental, Life, Natural Science and Technology, Okayama University, Okayama, 700-8530 Japan

**Keywords:** Vietnamese buffalo, White coat color, LINE1 insertion, *ASIP*

## Abstract

The coat color of the swamp buffalo is commonly dark gray, while the white coat color variant, which may have potential heat stress advantages, is also present in some Asian countries, including Vietnam. This study analyzed the most likely candidate genes, *ASIP* and *TYR*, responsible for the white coat color of Vietnamese native buffaloes. We found that LINE1 insertion in *ASIP*, a mutation previously reported in white swamp buffalo from other Asian countries, was exclusively found in white Vietnamese buffalo. Moreover, significantly higher expression of *ASIP* was confirmed in the hair follicles of white buffalo. On the other hand, no variants associated with the white phenotype were found in *TYR.* These findings indicate that the LINE1 insertion in *ASIP* is responsible for the white coat color in Vietnamese native buffalo, and that provides a crucial step towards their utilization and improved productivity in Vietnam.

**Supplementary Information:**

The online version contains supplementary material available at 10.1007/s11250-025-04309-7.

## Introduction

Water buffalo (*Bubalus bubalis*) are classified into two primary subtypes: river type and swamp type. Vietnamese native buffalo belong to the swamp type and are primarily used for draught power and meat production. Approximately 2.23 million buffaloes are raised in Vietnam in 2022, but their numbers are decreasing yearly mainly due to agricultural mechanization and urbanization. Despite this decline, buffalo meat consumption is higher than beef in Vietnam (Nguyen 2000), with production increasing by 147.7% between 2000 and 2021 (Vietnam General Statistics Office). Therefore, effectively utilizing Vietnamese native buffalo by enhancing their characteristics remains crucial.

**Fig. 1:**
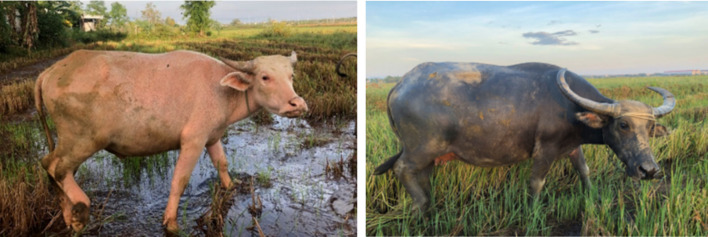
White and black coat colors of Vietnamese buffalo

The coat color of swamp buffalo is commonly dark gray, but a white coat color variant has also been reported in some countries, including Vietnam (Fig. 1). While buffaloes are adapted to hot and humid climates, their dark coat absorbs more solar radiation, potentially reducing productivity under heat stress (Marai and Haeeb 2010; Upadhyay et al. 2007). In contrast, lighter or white coats are known to mitigate these effects in cattle (Finch et al. 1984; Becerril et al. 1993; Maia et al. 2005). In Vietnam, up to 35% of native buffalo populations exhibit a white coat color in some areas (Berthouly 2008), yet the genetic factors underlying this trait remain unknown, despite its potential implications for breeding strategies in tropical climates.

Regarding genetic mutations causing white coat color, tyrosinase (*TYR*) is a well-known gene in humans and animals (Damé et al. 2012). In water buffalo, a nonsense mutation in *TYR* were reported in white individuals of Murrah buffalo (Damé et al. 2012). Recently, the insertion of a LINE1 transposon element into Agouti signaling protein (*ASIP*) was found to cause the white coat color of swamp buffalo from China, Bangladesh, and Thailand (Liang et al. 2021). This LINE1 insertion in *ASIP* could be the likely cause of the white phenotype in Vietnamese native buffalo. However, swamp buffalo have a strong geographic genomic diversity, and those in Vietnam were dispersed from the domestication area via different routes than those in China, Bangladesh, and Thailand (Zhang et al. 2016). Therefore, we considered the two possibilities: the Vietnamese white buffalo either inherited the LINE1 insertion in *ASIP* from a common ancestor with other Asian white buffalo or developed a new mutation in a gene such as *TYR* and *ASIP* after divergence. This study analyzed these genes in the Vietnamese native buffalo with white coat color to identify the causative mutation.

## Materials and methods

Hair follicle samples from 48 Vietnamese native buffaloes (41 black and seven white) were collected from 16 households in Thua Thien Hue province, Vietnam, ensuring minimal genetic relatedness based on farmer-provided lineages. All procedures were approved by the Animal Committee of Okayama University.

The LINE1 insertion in *ASIP* was detected by PCR using the primers from Liang et al. (2021). The PCR conditions were modified as follows: 94ºC for 2 min followed by 35 cycles of 94ºC for 30 s, 55ºC for 30 s, and 72ºC for 30 s. Genotypes were determined by electrophoresis of PCR products on 2% agarose gel.

The coding regions of *TYR* and *ASIP* in three white buffaloes were amplified by PCR and analyzed by Sanger sequencing. Identified missense mutations were subsequently genotyped in 48 buffaloes using the MinION Mk1C Nanopore sequencer (Oxford Nanopore Technologies). The passed reads were mapped to the *Bubalus bubalis* reference genome (NDDB_SH_1) using minimap2. Variant calling and genotyping were performed using the CLC Genomic Workbench software.

Total RNA was extracted from the hair follicles of five black and five white buffaloes. RT-qPCR was conducted with the Light Cycler 480 (Roche). The buffalo β-actin gene was used as an internal reference. Each assay was performed in triplicate. Relative mRNA expression was determined using the 2^−ΔΔCt^ method. Pairwise comparisons were conducted using Welch's t-test, assuming unequal variances between the two samples. The primers used in this study are listed in Table S1.

## Results

**Fig. 2:**
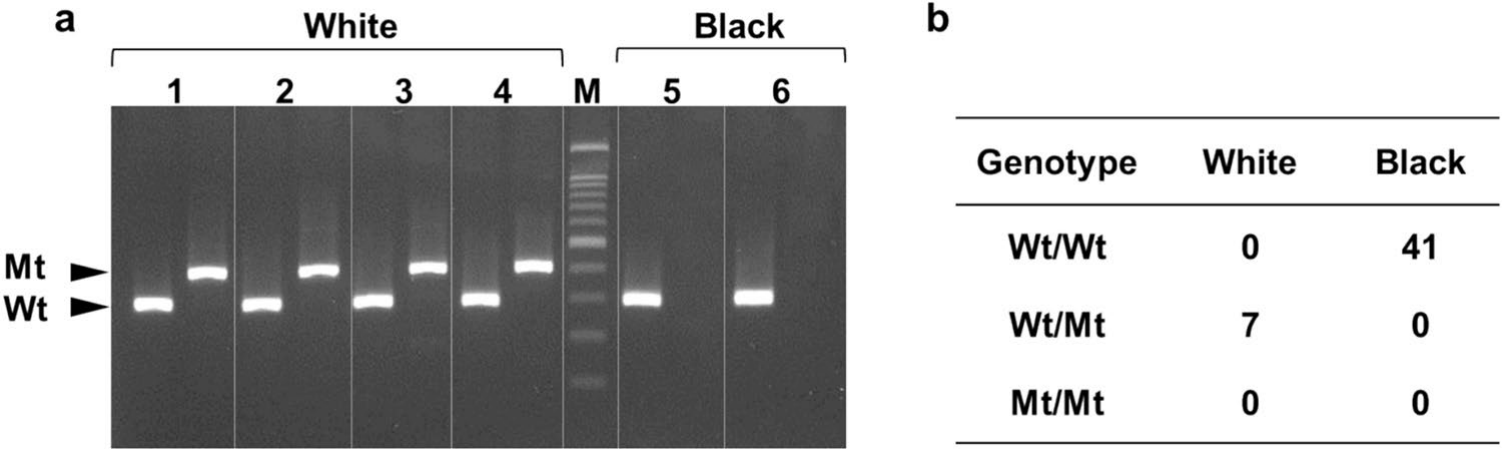
Genotyping of the LINE1 insertion in *ASIP* in Vietnamese buffalo. **a** Electrophoresis of PCR products of the LINE1 insertion, Wt: wild-type allele (296-bp), Mt: mutant-type allele (387-bp), M: 100-bp DNA ladder. **b** Genotype distribution of the LINE1 insertion in the buffalo population

**Fig. 3:**
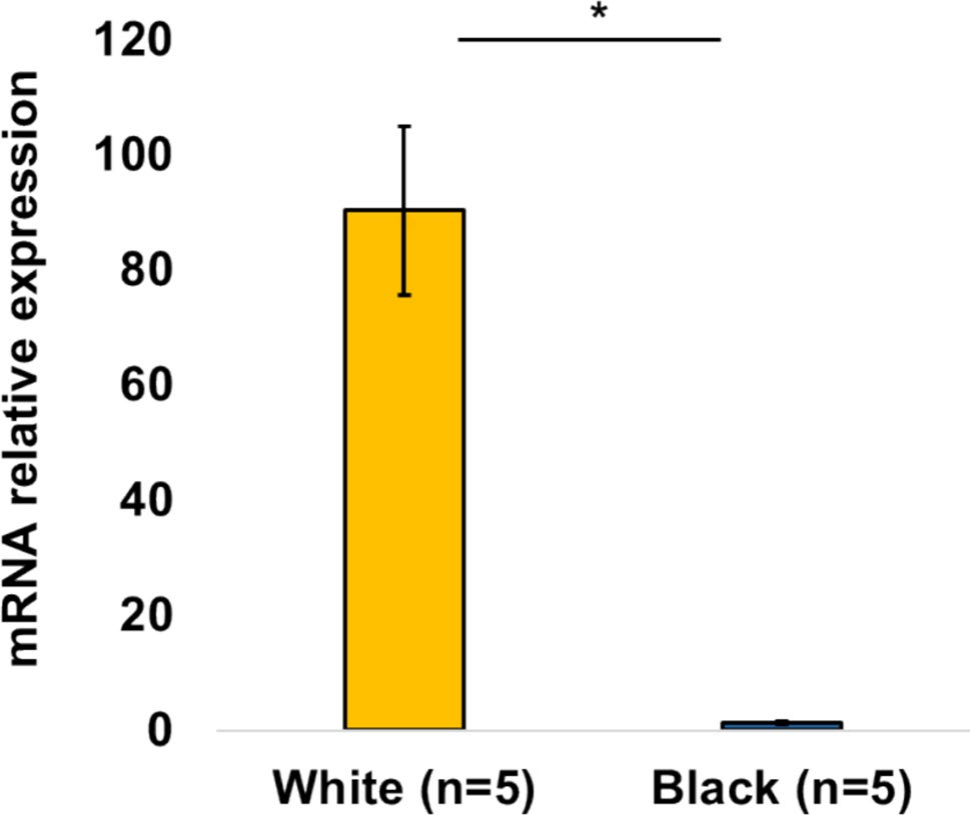
*ASIP* expression level in hair follicles of white (yellow) and black (blue) Vietnamese buffaloes. The data are presented as the mean ± SEM. * *p* < 0.01

As shown in Fig. 2, only a 296-bp DNA band from the normal sequences was detected in all the black buffaloes. In contrast, a 387-bp DNA band from the LINE1 insertion in *ASIP* was exclusively detected in all the white buffaloes. These results were consistent with those reported in white buffaloes from China, Bangladesh, and Thailand (Liang et al. 2021). Liang et al. 2021 reported a tenfold increase in *ASIP* mRNA expression in the skin of the white swamp buffalo due to the LINE1 insertion. To confirm this, we analyzed *ASIP* mRNA expression levels in the hair follicles of the Vietnamese white and black buffalo. Figure 3 shows a 70-fold higher *ASIP* expression in white buffalo than in black buffalo.

We found five novel nonsynonymous variants in *TYR* and none in *ASIP* from three white buffaloes (Table S2). Three (R255H, E319Q, I479T) in *TYR* were classified as either deleterious or probably damaging by one of the prediction tools. However, these variant alleles were presented in both white and black buffaloes, indicating no clear association between these variants and white coat color in Vietnamese buffalo (Table S2). Additionally, the known mutation reported in river buffalo was not found in these individuals. Thus, we concluded that the white coat color of Vietnamese native buffalo is attributed to the LINE1 insertion in *ASIP*.

## Discussion

The existence of white individuals in Vietnam has long been recognized, yet the genetic factors underlying this trait remained unclear until our study. In this study, we confirmed that the LINE1 insertion in *ASIP* was specifically found in Vietnamese white buffalo, while the novel mutation identified in *TYR* was not associated with the white coat color. Therefore, we established that the LINE1 insertion in *ASIP* is responsible for the white coat color in Vietnamese native buffalo.

Swamp buffalo is generally less productive in terms of milk and meat compared to river buffalo (Marai and Haeeb 2010). Interestingly, research has shown that under heat stress, cattle with lighter or white coat colors exhibit improved growth (Finch et al. 1984) and enhanced milk production (Becerril et al. 1993; Maia et al. 2005). These suggest that white swamp buffalo might provide unique economic advantages in tropical and subtropical regions. Notably, *ASIP* plays a role in fat metabolism (Sumida et al. 2004). Girardot et al. (2006) reported that brindle Normande cattle exhibited elevated expression of *ASIP* caused by a LINE1 insertion could be potential for enhancing milk and meat production. Japanese Black cattle with higher intramuscular fat levels exhibit over ninefold *ASIP* mRNA expression levels than Holstein cattle (Albrecht et al. 2012). Our study and Liang et al. 2021 found 70-fold and tenfold higher expressions of *ASIP* in the hair follicle and the skin of the white swamp buffaloes, respectively, indicating potential advantages of these white buffaloes in milk and meat production. Unlike river buffalo, where a nonsense mutation in *TYR* causes the white coat color (Damé et al. 2012), our study found no mutations in *TYR* linked to the white phenotype of Vietnamese native buffalo, reinforcing the uniqueness of the LINE1 insertion in *ASIP* in the swamp buffalo. If white swamp buffalo with this mutation demonstrate superior productivity in tropical regions, this trait could offer economic benefits not seen in river buffalo.

Our findings provide new insights into the genetic basis of white coat color in Vietnamese native buffalo. However, the small sample size of white individuals (n = 7) in this study may make it difficult to apply these results to all Vietnamese white buffalo. This limitation should be addressed in future studies by including a larger and more diverse population of white buffalo from different regions across Vietnam.

Given the expected rise in global temperatures, the potential advantages of white swamp buffalo in tropical regions warrant further investigation. Vietnam, where buffalo meat consumption exceeds beef and is increasing, is particularly relevant for these studies. Further research in areas with up to 35% white buffalo in Vietnam (Berthouly 2008) could provide valuable insights into their economic and environmental benefits. Although economic data on white swamp buffalo in Vietnam is lacking, our results offer a crucial step toward their utilization and improved productivity in Vietnam.

## Supplementary Information

Below is the link to the electronic supplementary material.

Supplementary file1 (DOCX 23 KB)

## Data Availability

The data is available from the corresponding author on reasonable request.
