# Supplementary material for: White coat color in Vietnamese native buffalo is attributed to the LINE1 insertion in ASIP
Source: Trop Anim Health Prod. 2025 Feb 11;57(2):54. doi: 10.1007/s11250-025-04309-7 (PMC11813964; doi:10.1007/s11250-025-04309-7)
Supplement: Supplementary file 1 — Supplementary file1 (DOCX 23 KB) [file 11250_2025_4309_MOESM1_ESM.docx]

**White coat color in Vietnamese native buffalo is attributed to the LINE1 insertion in *ASIP***

*Tropical Animal Health and Production*

Thuy Thanh Nguyen, Quan Viet Le and Takehito Tsuji

Corresponding author: Takehito Tsuji

Graduate School of Environmental, Life, Natural Science and Technology, Okayama University, Okayama 700-8530, Japan

Phone: + 81-86-251-8314, Fax: + 81-86-251-8388

E-mail: [takehito@okayama-u.ac.jp](mailto:takehito@okayama-u.ac.jp)

**Table S1** The list primers used in this study

| Name | Accession No. | Sequences (5′ to 3′) | Length (bp) | Ta^a^ (^o^C) |
| --- | --- | --- | --- | --- |
| TYR-Ex1 | NC_059161.1 | F: GACATTTTCTAGGCTTGGCAGGA  R: CTGGGTTTATGTAGATCGGATGG | 1262 | 56 |
| TYR-Ex2 |  | F: GGCCCAGGGTTTTGGATA AGAGT  R: CGAATAAGGAATCCCAACCTCCC | 382 | 58 |
| TYR-Ex3 |  | F: TGGACCTGTTGGCTTTTTGTACATC  R: TGCTCACTGGGTATCTAGAATATGAAC | 417 | 58 |
| TYR-Ex4 |  | F: TGGTCACATCAGACTGAGCAACTC  R: CTGACAGCATGAACTGTGGCTTTAC | 401 | 58 |
| TYR-Ex5 |  | F: GAGGACATCTTGAGGCAGGTACTTAG  R: CTTGGGGTTCTAGGTGTAACTTCCC | 1006 | 58 |
| ASIP-Wt | NC_059170.1 | F: TCGGAAACGACTAAAGTGAC^b^  R: CTCCAAAGATGATGTACTAATGAACAAT^b^ | 296 | 55 |
| ASIP-Mt |  | F: TTGTGGAATTTACTCGACGTT^b^  R: AAAGATTACCCACAGAAGGA^b^ | 387 | 55 |
| ASIP-Ex5,6 |  | F: CGAGATCAGGAAGGTTTTGGTAGC  R: CAGAGCACCAGCCCAAAGAAAC | 1787 | 60 |
| ASIP-Ex7 |  | F: CACCCCAACGTCAATAGCCAC  R: AAGTGGCTAGACTCCGAACC | 612 | 58 |
| ASIP-qPCR | XM_025263623.3 | F: TCTGTCTCTATCGTGGCACTG  R: TGCCACGTTCTTCATCGGAG | 108 | 60 |
| ACTB-qPCR | XM_025274489.3 | F: CATCGGCAATGAGCGGTTCC  R: ATACCGCAGGATTCCATGCCCAG | 75 | 60 |

^a^Annealing temperature.

^b^According to Liang et al. (2020).

**Table S2** Genotypes of identified nonsynonymous variants in *TYR* in Vietnamese buffalo

| Variant | Exon | Amino acid change | Prediction scores^a^ | | | Genotype | White (n=7) | Black (n=41) |
| --- | --- | --- | --- | --- | --- | --- | --- | --- |
|  |  |  | PolyPhen2  (0.5 - 1) | SIFT  (0 - 0.05) | PROVEAN  (≤ -2.5) |  |  |  |
| g.86963050C>T | 1 | V246I | 0 | 1 | -0.263 | V/V  V/I  I/I | 2  5  - | 35  5  1 |
| **g.86963022C>T** | 1 | **R255H** | 0.015 | 0.09 | **-2.966** | R/R  R/H  H/H | 1  5  1 | 16  24  1 |
| **g.86953313C>G** | 2 | **E319Q** | **1** | 0.24 | -1.59 | E/E  E/Q  Q/Q | 2  5  - | 11  22  8 |
| g.86953277A>G | 2 | S331P | 0.005 | 1 | -0.2 | S/S  S/P  P/P | -  -  7 | -  -  41 |
| **g.86853642A>G** | 5 | **I479T** | 0 | **0.04** | -1.674 | I/I  I/T  T/T | 5  2  - | 28  13  - |
| g.86853647C>T^b^ | 5 | W477X | - | - | - | W/W  W/X  X/X | 7  -  - | 41  -  - |

^a^The range scores of probably damaging or deleterious substitution are described in parentheses for each prediction tool. SNPs and prediction scores in bold were classified as “deleterious” or “probably damaging’’ by one of the prediction tools.

^b^Previously reported by Damé et al. (2012)
